# The Role of Non-penetrative Partnered Sex Activities in the Associations Among Erectile Difficulties, Sex and Relationship Satisfaction in Men Aged 50+

**DOI:** 10.1080/19317611.2023.2169850

**Published:** 2023-02-10

**Authors:** Anna Ševčíková, Jaroslav Gottfried, Veronika Gocieková, Gabriela Gore-Gorszewska, Lukas Blinka, Jan Kotík

**Affiliations:** a Psychology Research Institute, Faculty of Social Studies, Masaryk University, Brno, Czech Republic; b Institute of Psychology, Faculty of Philosophy, Jagiellonian University, Kraków, Poland

**Keywords:** Aging, sexual health, erectile difficulties, relationship satisfaction

## Abstract

**Objectives:**

This study examined the associations among erectile difficulties, sexual satisfaction, and relationship satisfaction, and whether there is a buffering effect of non-penetrative partnered sex on relationship satisfaction in men aged 50+ who report erectile difficulties.

**Methods:**

An analysis of survey data from 431 Czech men (aged 50–96, *Mdn* = 64).

**Results:**

Engagement in non-penetrative partnered sex did not moderate the relationship between erectile difficulties and sexual and relationship satisfaction, but it was linked to higher sexual and relationship satisfaction.

**Conclusions:**

The buffering effect of non-penetrative practices for the link between erectile difficulties and relationship satisfaction has not been supported.

## Introduction

Changes in sexual functioning among men after age 50 are significant and lead to a growing proportion of men who experience erectile difficulties (e.g., a 40% chance of having some form of erectile problems at age 40 with a 10% of increase per decade thereafter; Ferrini et al., 2017; Kaya et al., 2017; Quilter et al., 2017). Representative population-based studies inform that the prevalence of male sexual problems increases with age, with one-third of men aged 60+ reporting erection problems (Mitchell et al., 2013; Traen & Stigum, 2010). Regardless of their origin, erectile difficulties are linked to a decrease in sexual activity, particularly in penetrative sex (DeLamater et al., 2019; Ševčíková et al., 2021). Although penetrative sex remains the dominant and expected sexual activity in both middle-aged and older men and women (e.g., with 41 − 55% reporting intercourse in the past four weeks; Gore-Gorszewska, 2021; Lodge & Umberson, 2012; Mercer et al., 2013), sexual life and relationship satisfaction may be challenged due to an age-related onset of sexual difficulties as a barrier to pursuing penetrative sex (Karraker & DeLamater, 2013; Laumann & Waite, 2008; Lindau et al., 2007; Rosen et al., 2016).

According to studies about close relationships at mid and older age, sexual and relationship satisfaction are interconnected (Gillespie, 2017; Rahn et al., 2020; Rosen et al., 2016). Specifically, sexual satisfaction, which is a multi-faceted construct that includes the frequency of sexual activity as one of its components, may remain part of a satisfying relationship (Fleishman et al., 2020; Kontula & Haavio-Mannila, 2009; Smith et al., 2011; Stulhofer et al., 2010). Despite the fact that some couples report a certain level of sexual and relationship satisfaction, even when the frequency of penetrative practices decreases (Fileborn et al., 2017; Freak‐Poli & Malta, 2020; Traeen et al., *2019*), for many couples it is the opposite. A number of studies have documented that the onset of sexual problems is negatively associated with relationship satisfaction because, due to a decrease in sexual activity, people have the impression that losing a source of closeness and intimacy or a way used for buffering relationship, causes a conflict (Fallis et al., 2016; Rosen et al., 2016; Ševčíková & Sedláková, 2020).

Prior research has shown that erectile difficulty is a dominant topic in the sexual lives of middle-aged and older men (Fileborn et al., 2017; Lodge & Umberson, 2012; Ševčíková et al., 2021). This decreases sexual frequency and prompts various responses. There is evidence that men in these age groups tend to delay (or not seek) medical help, for a variety of personal reasons (Geerkens et al., 2020; Hinchliff et al., 2020, 2021; Štulhofer et al., 2020). If they overcome the potential (e.g., personal, cultural, social) barriers, they often contact their primary care physician or search for informal secondary sources (e.g., partner, friends, websites) (Geerkens et al., 2020; Hinchliff et al., 2020; Štulhofer et al., 2020).

However, not all erectile difficulties can be solved with medication (e.g., due to contraindication or after prostatectomy) and other coping mechanisms may be at play. Apart from assigning less importance to penetrative sex, which leads to a decline of the frequency of sex (Erens et al., 2019; Hillman, 2012; Lodge & Umberson, 2012; Riekkola et al., 2019; Wassersug et al., 2017), older people with health and sexual problems tend to reframe their understanding of sex by focusing on non-penetrative sexual activities, such as kissing, stroking, hugging, and, sometimes, oral sex (Connor et al., 2020; Erens et al., 2019; Freak‐Poli & Malta, 2020; Liu et al., 2019; Tetley et al., 2018; Waite et al., 2009). The use of the particular coping mechanism may depend on how partners’ sex lives were before the onset of erectile difficulties. It is more likely for this group of older men to prefer activities with which they are familiar and with which they feel comfortable (Erens et al., 2019). It also indicates that efforts to develop compensatory mechanisms occur more often in close relationships than in less close relationships. Partner support and the willingness to discover new, non-penetrative sexual opportunities were both listed as factors that promote non-coital activities (Wassersug et al., 2017). Either way, some studies indicate that using non-penetrative practices when struggling with erectile difficulties may be beneficial for maintaining sexual and relationship satisfaction (Connor et al., 2020; Tetley et al., 2018).

## Research aim

This study aims to examine the associations among erectile difficulties, satisfaction with sexual frequency (i.e., a component that represents sexual satisfaction, Štulhofer et al., 2010), and relationship satisfaction, and whether these associations differ for men aged 50+ who incorporate non-penetrative practices into their partnered sex life and those who do not or who do it to a lower extent. Upon prior research, we hypothesize the following relationships: Hypothesis 1a: There is a negative association between erectile difficulties and satisfaction with sexual frequency;

Hypothesis 1 b: There is a negative association between erectile difficulties and relationship satisfaction;

Hypothesis 2: There is a negative relationship between erectile difficulties and relationship satisfaction, which is mediated by satisfaction with sex frequency;

Hypothesis 3: The associations among erectile difficulties, satisfaction with the frequency of sex, and relationship satisfaction differ based on the level of engagement in non-penetrative practices;

Hypothesis 4a: Middle-aged and older men who use more non-penetrative activities are more satisfied with sex frequency than those who use non-penetrative practices to a lower extent;

Hypothesis 4 b: Middle-aged and older men who use more non-penetrative activities are more satisfied with their relationship than those who use non-penetrative practices to a lower extent.

## Materials and methods

### Participants and recruitment

The convenient sample included respondents who participated in an online survey on sexual life and intimate relationships in mid and later life. An online questionnaire published on the Qualtrics platform was advertised from December 2018 to February 2019 via social media and two dominant advertising agencies—Seznam.cz and Czech News Center (CNC)—that possess internal mechanisms to target advertising to Czech internet users aged 50 and older. Seznam.cz represents a web portal with over 30 associated web services, which is used by half of the Czech internet population. The marketing portfolio of CNC includes about 40 online newspaper and magazine products and it reaches about six million internet users (the Czech Republic has about seven million internet users aged 16 and over; CZSO , 2018). As an incentive to participate, respondents were offered a chance to win one of five total €40 gift vouchers. This study was approved by the authors’ Institutional ethical board.

The questionnaire was originally completed by 799 Czech respondents. A number of respondents were excluded due to the following hierarchically applied exclusion criteria: being female (*n* = 298); not being in a relationship (*n* = 35); and not having the opportunity for sexual intercourse (*n* = 35). After applying all of the exclusion criteria, the final sample consisted of 431 Czech males, aged 50–96 years old (*M* = 63.55, *SD* = 8.19, *Mdn* = 64, 23 participants reported a non-heterosexual orientation).

### Measurement

#### Relationship satisfaction

We used a four-item version of the *Couples Satisfaction Index* (Funk & Rogge, 2007). Respondents rated statements such as “*How rewarding is your relationship with your partner?*” on a Likert-type scale that ranged from 1 to 7, with higher values indicating higher satisfaction (i.e., from *Extremely unrewarding* (1) to *Extremely rewarding* (7)). The *Couples Satisfaction Index* showed very high internal consistency (Cronbach’s *α* = .95, McDonald’s *ω* = .96). For each respondent, we calculated the relationship satisfaction score as the mean of the item scores.

#### Satisfaction with the frequency of sex

To inquire about the respondent’s satisfaction with the frequency of sex, we designed a single item: “*How would you rate your satisfaction with the frequency of sex, i.e., how often did you have it in the last 12 months?*” The respondent’s score on this variable equaled the response to the aforementioned question on a Likert-type scale that ranged from *Very dissatisfied* (1) to *Very satisfied* (5). Although this item did not define sex, the question on satisfaction with sex frequency was administrated after the list of diverse sexual practices (see below: *Non-penetrative partnered sexual practices*).

#### Frequency of sexual intercourse

This variable indicates the frequency of sexual intercourse over the preceding 12 months and it was measured by a single item. The item read: “*In the last 12 months, how often have you had sexual intercourse?*”. “Sexual intercourse” is the translation of the Czech term “pohlavní styk” that was employed in the questionnaire and is commonly used in the Czech language, referring to genital penetrative sex (mostly vaginal intercourse). In order to better fit a linear trend on the original non-linear interval scale, we assigned numbers to each interval to indicate the probable lowest frequency of sexual activities per month: *Not once* (0), *Less than once a month* (0.5), *Once a month* (1), *Two or three times a month* (2.5), *Once per week* (4), and *Several times a week* (8).

#### Erectile difficulties

We measured erectile difficulties using the International Index of Erectile Function (IIEF-5, Rosen et al., 1999), the Czech version of which is commonly used by Czech sexologists and urologists (Broul & Schraml, 2011). Using three items, respondents were asked to assess their ability to achieve and maintain an erection (e.g., “*When you were sexually stimulated to get an erection, how often was your erection sufficient for sexual intercourse?”, “How difficult is it for you to maintain your erection till the end of sexual intercourse?”*). The items offered a Likert-type answer scale that ranged from 1 to 5, with the higher number indicating more serious or frequent problems with achieving and retaining an erection. We calculated the erectile difficulties score as the mean answer score.

#### Non-penetrative sexual practices

Non-penetrative sexual practices is a dichotomous variable that indicates either practicing non-penetrative practices less frequently or never (0) or practicing them more frequently (1). To create this variable, we used three items to ask the respondents about the frequency of their participation in petting, touching their partner’s genitals, and oral sex, in the preceding 12 months. Respondents indicated the frequency of each activity on a scale with answer options: *Not even once* (1), *Less than once a month* (2), *Once a month* (3), *Twice or three times a month* (4), *Once a week* (5), or *Several times a week* (6). Respondents who indicated the frequency of any activity as *Once a week* (5) or *Several times a week* (6) were assigned a score of 1 for this variable; the rest were assigned a score of 0, referring to the less frequent or lacking frequency. We chose the cutoff score 4/5 based on the median split and the distribution of respondents’ maximum scores, which was strongly negatively skewed (modus = 6). The cutoff score 4/5 also allowed for keeping an acceptable group size ratio of roughly 2:1 in favor of the more sexually active group.

The main reason for dichotomizing the originally continuous variable was that we used it as a moderator variable in a complex model. This is better achieved by inspecting model invariance between two groups based on a categorized variable than by specifying one large model with multiple interaction terms that use the original continuous variable. This allowed us to gain an insight into the correlations and descriptive statistics for each group separately. Additionally, this approach is potentially more forgiving in terms of penalties to the statistical power of the tests, due to the multiple interaction terms included in the model. However, because of the categorization, we could not estimate linear continuous trends for changes in non-penetrative sexual practices, but only the differences between two groups of people.

### Analyses

Data analyses were conducted in R (R Core Team, 2019b). We used the *foreign* package (R Core Team, 2019a) to load the data, the *tidyverse* package (Wickham, 2017) to clean the data, the *psych* package (Revelle, 2019) to inspect the data, the *mediation* package (Tingley et al., 2014) for mediation analysis, and the *lavaan* package (Rosseel, 2012) for the path model analysis.

**Figure 1.**
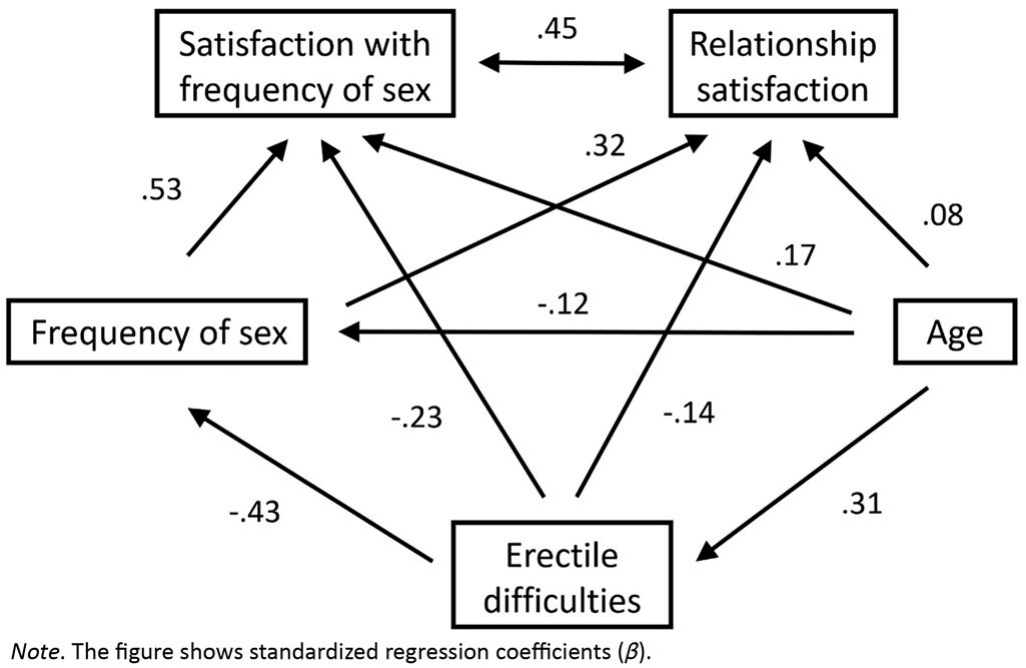
The associations among erectile difficulties, satisfaction with sexual frequency, and relationship satisfaction. *SEM path diagram.*

**Figure 2.**
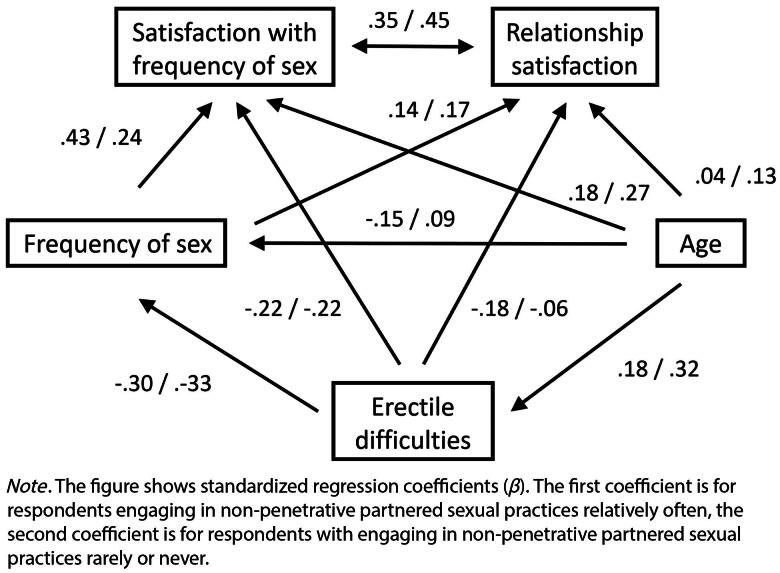
The associations among erectile difficulties, satisfaction with sexual frequency, and relationship satisfaction with a grouping variable for non-penetrative partnered sexual practices. *SEM path diagram for two sexual practices groups.*

We used structural equation modeling (SEM) path analysis to test Hypotheses 1a and 1 b (see Figures 1 and 2) and we tested model invariance with regard to non-penetrative practices to find support for Hypothesis 3. We used mediation analysis to test Hypothesis 2 with relationship satisfaction as the dependent variable, erectile difficulties as the predictor, and satisfaction with the frequency of sex as the mediator. To estimate the mediation effect, we used the nonparametric bootstrap (*n* = 2000) method. We expected that a negative relationship between erectile difficulties and relationship satisfaction would be mediated by satisfaction with sex frequency. In addition, we used independent-sample t-tests to assess the differences between non-penetrative sexual-practice groups regarding their satisfaction with the frequency of sex and relationship satisfaction, as per Hypotheses 4a and 4 b. The level of significance for all analyses was set to *p* = .01.

## Results

### Descriptive statistics

Descriptive statistics are presented in Table 1. About 59% (258) respondents indicated that they engaged in non-penetrative practices at least once a week, 34% (146) respondents indicated a lower frequency, and 7% (30) respondents did not answer the question. Bivariate relationships between the variables are shown in Table 2 via correlation matrix.

**Table 1.** Descriptive statistics.

	*n*	*M*	*SD*	Mdn	Min	Max
Age	431	63.55	8.19	64	50	96
Satisfaction with sex frequency	391	3.31	1.21	4	1	5
Relationship satisfaction	350	5.30	1.35	5.5	1	7
Frequency of sex (per month)	422	3.76	2.61	4	0	8
Erectile difficulties	377	2.30	0.96	2	1	5

**Table 2.** Pairwise correlation matrix.

	1	2	3	4	5
1. Age	1.00				
2. Satisfaction with sex frequency	−0.04	1.00			
3. Relationship satisfaction	−0.05	0.56*	1.00		
4. Frequency of sex (per month)	−0.26*	0.60*	0.37*	1.00	
5. Erectile difficulties	0.32*	−0.43*	−0.27*	−0.47*	1.00

*Note. N* = 431. *p < .01.

To test Hypotheses 1a and 1 b (on negative associations between erectile difficulties and both satisfaction with sexual frequency and relationship satisfaction), we constructed a SEM path model. Even after controlling for the effects of other variables and their covariance, there was a weak negative effect for erectile difficulties on relationship satisfaction, *B* = −0.20, *SE* = 0.07, *p* = .006, as well as for satisfaction with the frequency of sex, *B* = −0.29, *SE* = 0.08, *p* < .001. See Figure 1 for a full solution, with standardized coefficients. Thus, we found support for both hypotheses.

To test Hypothesis 2 (i.e., a negative relationship between erectile difficulties and relationship satisfaction is mediated by satisfaction with sex frequency), we employed a mediation model. Based on a post-hoc power analysis and given *n* = 327 complete observations, we achieved sufficient statistical power (*α* = .01, *β* = .05) to detect small mediation effects (smallest expected effect: *r* = −0.3).

We found that, out of the total effect of the erectile difficulties on the relationship satisfaction (*B* = −0.41, 95% CI [−0.55, −0.26], *p* < .001), roughly 76% (95% CI[49%, 100%]) could be explained as an indirect effect of erectile difficulties on the relationship satisfaction as mediated by the satisfaction with the frequency of sex (*B* = −0.31, 95% CI [−0.41, −0.22], *p* < .001). This finding suggests that erectile difficulties could negatively affect relationship satisfaction through dissatisfaction with the frequency of sex, providing indirect correlational support for Hypothesis 2. The strength of the mediation effect can be described as weak to moderate.

To test Hypothesis 3 (i.e., the relationship between erectile difficulties and relationship satisfaction, and the satisfaction with the frequency of sex differs based on non-penetrative sexual practices), we constructed SEM path models (Figure 2) with non-penetrative practices as a grouping/moderating dichotomous variable (due to missing answers on the grouping variable, *N* = 401). In the model, we also controlled for the effects of other predictors (e.g., age, the frequency of sex). For people engaging in non-penetrative activities less frequently or not at all, we found no significant differences in the relationship of erectile difficulties with relationship satisfaction, Δ*B* = 0.19, *SE* = 0.17, *p* = .268, or with satisfaction with the frequency of sex, Δ*B* = 0.06, *SE* = 0.17, *p* = .708, compared to people engaging in non-penetrative activities often. The above-reported effects are essentially the differences in unstandardized regression coefficients between the two groups. Since these differences are not statistically significant, the data provide no support for Hypothesis 3. Figure 2 provides a model solution with standardized regression coefficients for each group.

*T*-tests were used in order to test Hypotheses 4a and 4b (i.e., men who use more non-penetrative activities are more satisfied with sex frequency and their relationship than those who use non-penetrative practices to a lower extent). We found that men who often engaged in non-penetrative practices were much more satisfied with the frequency of the sex, *t*(260) = −11.14, *p* < .001, Cohen’s *d* = −1.22, 95% CI [−1.45, −1.00] than men who engaged in these practice less frequently or never. Men who often engaged in non-penetrative practices were also much more satisfied in their relationships, *t*(198) = −7.76, *p* < .001, Cohen’s *d* = −0.95, 95% CI [−1.18, −0.71]. These test results provide strong support for Hypotheses 4a and 4b. Men who often engage in non-penetrative practices were slightly younger (*p* < .001, Cohen’s *d* = 0.40), engaged in sex much more often (*p* < .001, Cohen’s *d* = −1.45), and experienced considerably less serious erectile difficulties (*p* < .001, Cohen’s *d* = 0.86). Even with non-normal variable distribution in some cases, all differences can be considered reliable. Overall, both groups significantly differ in all of the aforementioned variables with generally large effect sizes, as seen in Table 3.

**Table 3.** Differences between groups in sexual practices.

	NPPSP rarely or never		NPPSP often		*t*	*df*	*p*	Cohen’s *d*
	*M*	*SD*	*M*	*SD*				
Age	65.38	8.08	62.19	7.74	3.86	291	<.001	0.40
Satisfaction with sex frequency	2.54	1.09	3.80	0.99	−11.14	260	<.001	−1.22
Relationship satisfaction	4.56	1.41	5.71	1.09	−7.76	198	<.001	−0.95
Frequency of sex (per month)	1.87	1.71	4.95	2.34	−15.14	376	<.001	−1.45
Erectile difficulties	2.75	1.04	2.02	0.74	7.20	207	<.001	0.86

*Note*. NPPSP: non-penetrative partnered sexual practices.

## Discussion

The study aimed to examine the associations among erectile difficulties, satisfaction with sexual frequency, and relationship satisfaction. It also examined whether these variables and their associations differ based on the extent of non-penetrative partnered sexual practices among men aged 50+.

The results show that erectile difficulties are negatively linked to both the satisfaction with sexual frequency and relationship satisfaction. Notably, these associations were observed to be similar regardless of the frequency of non-penetrative partnered sexual activities. This suggests that erectile difficulties in mid and later life may put additional strain on couples and their relationship satisfaction. Although non-penetrative sexual activities are known to be used for maintaining sexual and relationship satisfaction (Connor et al., 2020; Tetley et al., 2018; Wassersug et al., 2017), our findings do not support the notion that they have the potential to buffer the negative effects of erectile difficulties on sexual or relationship satisfaction (Ayalon et al., 2019; Fileborn et al., 2017; Gewirtz-Meydan et al., 2019). An explanation could be that, despite the potential benefits of non-coital activities, some men aged 50+ may still prioritize penetrative sex, with intercourse considered to be the gold standard and a sign of manhood and masculinity (DeLamater, 2012; Gore-Gorszewska, 2021; Lodge & Umberson, 2012). Specifically, in men who tend to perceive non-penetrative sexual activities primarily as foreplay and who expect penetrative sex to follow, the potential buffering effect might not emerge. It is possible that our sample had respondents who prioritize penetrative sex and who might perceive non-coital sexual practices to be only a prerequisite, or an unsatisfying alternative for sexual intercourse when faced with barriers induced by the onset of erectile difficulties. This interpretation could be supported by the fact that penetrative intercourse is the preferred form of sex among the Czech older population, and dominates other sexual activities, such as masturbation (Steklíková, 2014). In line with this, participants’ partners may insist on pursuing penetrative sex instead of non-coital practices (Gewirtz-Meydan et al., 2019; Lodge & Umberson, 2012).

An interesting finding, however, is that men who used non-penetrative partnered sexual practices more often reported fewer erectile difficulties, greater satisfaction with sexual frequency, and more relationship satisfaction compared to the group of men who did not use them at all or who used them less frequently. The simplest and most straightforward explanation could be that the former group was slightly younger and thus probably healthier (Schick et al., 2010). Nonetheless, the age gap between the groups—less than 3 years, on average—was too small to provide a sufficient explanation. An alternative explanation could be that performing non-coital sexual activities can make erectile difficulties less distressing or less pronounced, like when preceding intercourse with non-penetrative sexual activities may facilitate achieving erection despite moderate erectile difficulties. This could potentially account for a lower number of erectile difficulties, and higher sexual and relationship satisfaction as reported by the participants who often engage in non-coital sex. According to previous research, there is a positive association between variability in sexual practices and sexual satisfaction (Gillespie, 2017). This linkage is often explained with open sexual communication (Gillespie, 2017), which is generally beneficial, not only for sexual and relationship satisfaction, but also for addressing emerging sexual problems (Erens et al., 2019; Štulhofer et al., 2019; von Humboldt et al., 2021). However, another explanation might also be at play: some people who experience more serious erectile difficulties might cease their sexual life (i.e., stop engaging in both sexual intercourse and non-penetrative sexual activities; Wassersug et al., 2017; Lodge & Umberson, 2012). In other words, our observation that people who often engage in non-penetrative practices show less serious erectile difficulties might not be the result of practices to alleviate erectile difficulties, but instead show that people with more serious erectile difficulties cease their participation in such practices. Though our design does not allow us to make causal claims in this regard, the results indirectly imply the beneficial effect of engaging in a wider range of sexual techniques on relationship and sexual satisfaction.

This study also showed that satisfaction with sexual frequency has a moderately strong positive association with relationship satisfaction, which suggests that the quality of sexual life remains a relevant aspect of relationship functioning, even in men aged 50+ (Fallis et al., 2016; Fleishman et al., 2020; Geerkens et al., 2020; Kouidrat et al., 2017; Rosen et al., 2016). However, since this study was based on a convenience sample, the generalizability of our findings to the general population of heterosexual men in mid and later life may be limited. Moreover, the participants of this study were predominantly sexually active. Therefore, they did not face a steep sexual decline that would negatively impact their sexual and relationship satisfaction and lead to a reinterpretation of the importance of sex for relationship functioning (a phenomenon described in qualitative studies on later-life sexuality; Fileborn et al., 2017; Gore-Gorszewska, 2021; Gott & Hinchliff, 2003; Ševčíková & Sedláková, 2020).

The mediation model provides modest support for the idea that a negative relationship between erectile difficulties and relationship satisfaction could be mediated by satisfaction with sex frequency. Erectile difficulties in men aged 50+ may impact satisfaction with sex frequency that, in turn, could affect relationship satisfaction. However, this finding, including the interpretation, should be taken with caution because the mediation model was tested on cross-sectional data and it might be more plausible that all three variables share a certain portion of variance, rather than causally affect each other. In addition, satisfaction with sex frequency was not explicitly defined so that some of the participants could interpret this item as satisfaction with the frequency of penetrative sex only. Nonetheless, it is still worth mentioning that the analyses were built on the assumption that erectile difficulties can affect both sexual and relationship satisfaction. While this assumption is supported by previous research (Lee et al., 2016; Tong & Waite, 2020), the reverse causal link might be at play in that relationship satisfaction may negatively affect sexual functioning, like when the partners’ views on sexuality diverge or when there is an absence of partner support in solving sexual problems (Boddi et al., 2015; Gillespie, 2017).

It also should be noted that the study findings may be limited by the fact that our sample included men in both middle age and late adulthood. Possible differences between these two populations could not be elaborated further due to the small number of participants at an advanced age. The scores for the studied satisfaction constructs were high, so it seems likely that, possibly out of curiosity, satisfied men participated in this online survey. That may warrant caution in generalizing the findings of this study.

## Conclusion

This study showed that erectile difficulties in mid and later life are negatively related to satisfaction with sexual frequency and relationship satisfaction. However, the correlation between erectile difficulties and relationship satisfaction and the satisfaction with the frequency of sex was found to be stable regardless of non-penetrative partnered practices. In other words, more frequent engagement in non-penetrative sexual practices does not seem to interact with the assumed negative effect of erectile difficulties in sexual and relationship satisfaction. However, a wider sexual repertoire might be beneficial for sexual functioning among men in later life, because it was associated with a lower incidence of erectile difficulties. These findings might have an implication for clinicians who, when working with middle-aged and older clients with erectile difficulties, often advise them to incorporate non-penetrative partnered sexual activities as a solution to their problems. This study results suggest that the situation is more complex and attention should be paid to how these men understand sexual practices, particularly non-coital partnered activities, and in which context and what meanings they ascribe to them.
